# Improvement of the design and generation of highly specific plant knockdown lines using primary synthetic microRNAs (pri-smiRNAs)

**DOI:** 10.1186/1756-0500-3-59

**Published:** 2010-03-04

**Authors:** Sandra Niemeier, Leonardo Alves, Thomas Merkle

**Affiliations:** 1 Institute of Genome Research and Systems Biology, RNA-based Regulation, Faculty of Biology, University of Bielefeld, D-33594 Bielefeld, Germany; 2 Current address: John Innes Centre, Dept. of Cell and Developmental Biology, Norwich Research Park, Colney, Norwich NR4 7UH, UK

## Abstract

**Background:**

microRNAs (miRNAs) are endogenous small non-coding RNAs that post-transcriptionally regulate gene expression. In plants, they typically show high complementarity to a single sequence motif within their target mRNAs and act by catalyzing specific mRNA cleavage and degradation. miRNAs are processed from much longer primary transcripts via precursor miRNAs containing fold-back structures. Leaving these secondary structures intact, miRNAs can be re-designed experimentally to target mRNAs of choice.

**Results:**

We designed primary synthetic miRNAs (pri-smiRNAs) on the basis of the primary transcript of the Arabidopsis *MIR159A *gene by replacing the original miR159a and the corresponding miR159a* with novel sequences, keeping the overall secondary structure as predicted by the program *RNAfold*. We used the program *RNAhybrid *to optimize smiRNA design and to screen the complete Arabidopsis transcriptome for potential off-targets. To improve the molecular cloning of the pri-smiRNA we inserted restriction sites in the original *MIR159A *primary transcript to easily accommodate the smiRNA/smiRNA* DNA fragment. As a proof-of-concept, we targeted the single gene encoding chalcone synthase (CHS) in Arabidopsis. We demonstrate smiRNA(CHS) expression and *CHS *mRNA cleavage in different transgenic lines. Phenotypic changes in these lines were observed for seed color and flavonol derivatives, and quantified with respect to anthocyanin content. We also tested the effect of mismatches and excess G:U base pairs on knockdown efficiency.

**Conclusions:**

*RNAhybrid*-assisted design of smiRNAs and generation of pri-smiRNAs using a novel vector containing restriction sites greatly improves specificity and speed of the generation of stable knockdown lines for functional analyses in plants.

## Background

microRNAs (miRNAs) are 21-24 nucleotide (nt) long, endogenous non-coding RNA molecules that are involved in the post-transcriptional regulation of gene expression in multicellular eukaryotic organisms. miRNAs in animals and plants differ not only in their biogenesis [1] and in their distribution within the genome [2,3], but also in their preferred mode of action. In plants, miRNAs typically show near-perfect complementarity to a single sequence stretch in the coding region of their target transcripts [4]. This allows a very specific recognition of the target mRNA by the miRNA, which is incorporated into a large protein complex, the RNA-induced silencing complex (RISC) that mediates the cleavage of the target mRNA. In animals, however, usually several binding sites for a miRNA are found in the 3'untranslated region (3'UTR) of the target transcripts [5] and the complementarity between the miRNA and their targets is much lower and often restricted to the 5' part of the miRNA:mRNA hybrid [6]. Typically, the translation of the target mRNA is repressed by RISC and the complex is most probably transported to the so-called processing bodies, where the target mRNA is first stored and then degraded [7]. However, there are still open questions dealing with the structural requirements of miRNA:mRNA hybrid structures concerning target selection and mode of function in plants and animals [8]. In addition, new work revealed that there is a higher degree of miRNA-based translational repression in plants than anticipated before [9].

Different approaches like genetic screening, cloning of small RNAs, *in silico *prediction methods or the use of deep sequencing technologies have been applied for the identification of new miRNAs [2,10-17]. The main characteristics of miRNAs to distinguish them from other small RNAs are their length of 21 to 24 nt, processing by DCL1 from endogenous primary transcripts (pri-miRNAs) containing foldback structures, the low minimum free energy (mfe) value of the precursor miRNAs (pre-miRNAs), and the existence of mRNA targets [2,18]. Primary transcripts of miRNA genes can be considerably longer and may have a much more complex structure than the pre-miRNA. Interestingly, the stem-loop structure or shape of the pre-miRNA is more important for the correct biogenesis of the mature miRNA than the miRNA sequence itself [19]. This feature of miRNAs offers the possibility to create synthetic or artificial miRNAs (smiRNAs or amiRNAs) to target genes of interest by exchanging only the miRNA and miRNA* sequences in a known pre-miRNA or pri-miRNA without changing the stem-loop structure [19-22]. This opened a novel approach for the functional characterization of genes and gene families in Arabidopsis and in other plants. The use of synthetic miRNAs is the method of choice for the generation of highly specific and stable knockdown lines, since specificity can be much better controlled as with RNA interference (RNAi) methodologies. Here, we show that the design of smiRNAs can be optimized by using the miRNA target prediction program *RNAhybrid *[23] in order to minimize off-targets. In addition, we optimized the cloning of pri-smiRNAs which can now be generated in a one-step procedure. As proof-of-principle, we applied this procedure to generate efficient knockdown lines for the gene encoding chalcone synthase (CHS) in Arabidopsis.

## Results

### Design of synthetic primary-microRNAs (pri-smiRNAs)

We designed a smiRNA to target transcripts of the *CHS *gene that encodes a key enzyme of the flavonoid biosynthesis pathway [24,25]. In Arabidopsis, CHS is encoded by a single gene, and *chs *knockout mutants show easily detectable phenotypes in seeds and in seedlings [26].

**Figure 1:**
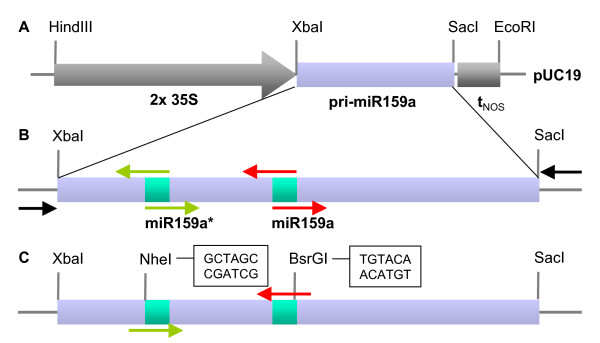
**Improvement of the generation of pri-smiRNAs for knockdown lines**. **(A) **Backbone of the endogenous pri-miR159a driven by the strong 35S promoter. The expression cassette flanked by the *HindIII *and *EcoRI *sites of the pUC19 vector was moved into the binary vector pGPTV-BAR for the generation of transgenic lines. **(B) **Standard procedure to create a pri-smiRNA by exchanging miR159 with smiRNA and miR159* with smiRNA* sequences, respectively, by a two-step procedure involving overlap-extension PCRs. **(C) **"Easy cloning vector" (ECV) procedure that allows the generation of any pri-smiRNA by a one-step PCR-cloning procedure due to the introduction of restriction sites. Primers required for each procedure are indicated by arrows. Vector primers are in black. The sequences of the introduced restriction sites are given as inserts.

Different candidate smiRNAs designed to target *CHS *transcripts were tested *in silico *with the program *RNAhybrid *to optimize the minimal free energy (mfe) of the smiRNA:mRNA hybrid structure [23]. Also using the program *RNAhybrid*, smiRNA candidates were tested *in silico *against the complete Arabidopsis transcriptome (See additional file 1: *In silico *target predictions for smiRNA(CHS) using the program *RNAhybrid*). Occurrence of additional undesired targets (off-targets) were minimized by changing and/or shifting the sequence of a smiRNA candidate relative to the mRNA target and re-testing the new smiRNA candidate against the Arabidopsis transcriptome again. The smiRNA that was finally chosen to target the *CHS *gene, termed smiRNA(CHS), had no mismatches to its target. In addition, we identified only one potential off-target. The mfe value for the smiRNA(CHS):*CHS *mRNA hybrid structure is well below -30 kcal/mol, as is the case with the majority of validated plant miRNA:mRNA hybrids [27]. The sequences of the smiRNA(CHS) and the corresponding smiRNA*(CHS) were inserted into the primary transcript of *MIR159A *via two consecutive overlap extension PCRs [28], referred to as standard procedure. Thereby the original miR159a and miR159a* sequences in the pri-miR159a were replaced by smiRNA(CHS) and the corresponding smiRNA* (Figure 1A, B), similarly to the method described by [21]. pri-miR159a was chosen as a backbone for our smiRNA approach because it is the primary transcript of a well-documented miRNA gene that produces high levels of mature miRNAs [2]. The secondary structures of the candidate pri-smiRNAs were analyzed with the program *RNAfold *[29]. To avoid possible differences in processing, care was taken that the predicted secondary structure of the pri-smiRNA(CHS) was identical to the structure of the wild type pri-miR159a. To this end, the 2-nt-mismatch (loop) within the original miR159a:miR159a* hybrid structure was also engineered into the pri-smiRNA(CHS) (See additional file 2: Sequences and predicted secondary structures of pri-smiRNAs using the program *RNAfold*).

To simplify pri-smiRNA cloning, we designed an "easy cloning vector" (ECV) consisting of pri-miR159a containing restriction sites flanking the precursor miRNA sequence (Figure 1C). The predicted secondary structure of this pri-smiRNA(CHS) ECV contains a small extra loop outside the precursor secondary structure (See additional file 2: Sequences and predicted secondary structures of pri-smiRNAs using the program *RNAfold*). In contrast to the two-step procedure, however, pri-smiRNAs of choice can now be generated in a one-step procedure using a standard PCR and only two instead of six primers (Figure 1). We also generated transgenic pri-smiRNA(CHS) ECV lines and compared them with the pri-smiRNA(CHS) lines generated with the standard procedure.

### Molecular analyses of transgenic lines expressing pri-smiRNA (CHS)

**Figure 2:**
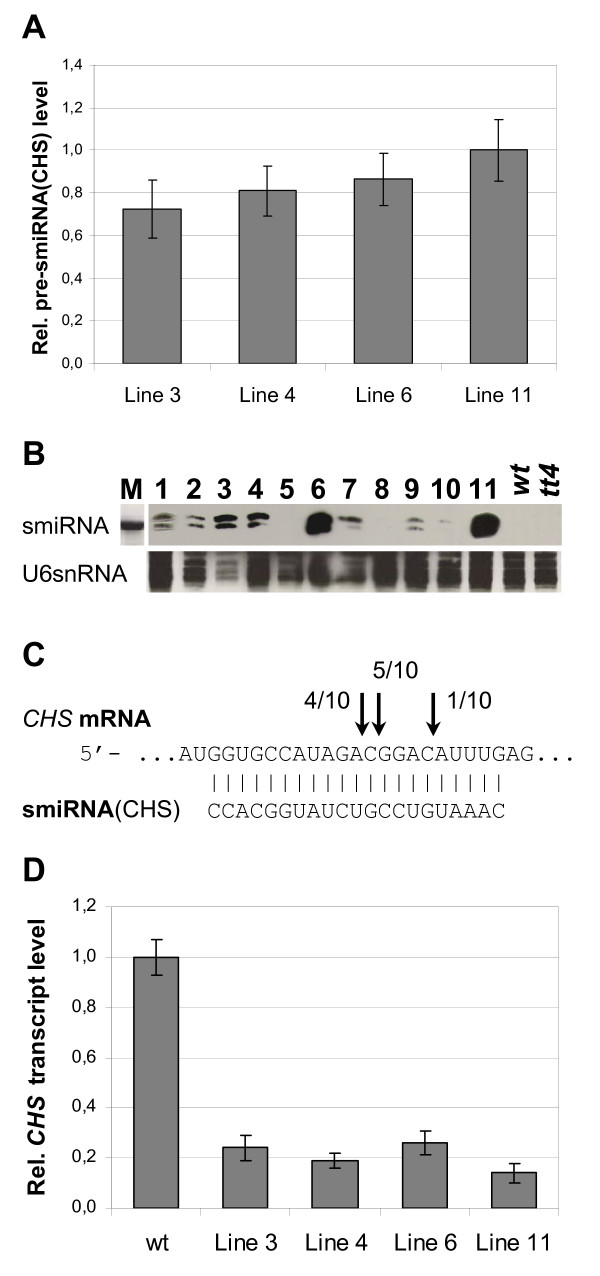
**Molecular analyses of transgenic lines expressing pri-smiRNA(CHS)**. **(A) **Quantitative analysis of the relative transcript levels of the smiRNA(CHS) precursor by qRT-PCR experiments (RNA from wild type seedlings gave no signal) **(B) **RNA extracted from transgenic pri-smiRNA(CHS) lines numbered 1-11 was used for small RNA Northern blots to detect smiRNA(CHS) production. RNA from wild type (*wt*) and *chs *knockout (*tt4*) plants served as control. The upper panel shows signals obtained with the smiRNA(CHS) probe, the signals in the lower panel were obtained using a probe for U6snRNA as loading control. Size marker (M): 21 nt-long RNA oligonucleotide. **(C) **Summary of validation experiments using RLM-5'RACE experiments. The smiRNA(CHS):*CHS *mRNA hybrid is shown. Arrows and numbers refer to the positions and relative abundance of 5'RACE products analyzed. **(D) **Quantitative analysis of the *CHS *mRNA target by qRT-PCR experiments. Data in (A, D) were normalized to the highest values. The same transgenic lines were also analyzed in Figure 3.

The expression of the pre-smiRNA(CHS) from the modified pri-miR159a backbone and the processing to smiRNA(CHS) was assayed in transgenic Arabidopsis lines. pre-smiRNA(CHS) was detected by quantitative reverse-transcription real time PCR (qRT-PCR) experiments using RNA extracted from different transgenic lines, but not in RNA from wild type plants (Figure 2A). The expression level of pre-smiRNA(CHS) varied considerably in different transgenic lines. Similarly, the level of the smiRNA(CHS) that was detected by small RNA Northern blots showed corresponding variations. Using probes specific for smiRNA(CHS), we detected signals in lanes containing RNA from plants over-expressing pri-smiRNA(CHS) but not in lanes containing RNA from wild type Arabidopsis seedlings (Figure 2B). Two small RNA fragments were detected, one 21 nt in length, the other one 22 or 23 nt long. The effect of smiRNA(CHS) expression on its target was assayed as well. First, validation of target cleavage products that were induced by the expression of the smiRNA was performed using a modified RNA ligase-mediated rapid amplification of cDNAs ends (RLM-5'RACE) approach, which is used to precisely map the position of the cleavage induced by the RISC complex [10]. In nine out of ten 5'RACE products analyzed, cleavage of *CHS *mRNA was detected in the middle of the smiRNA(CHS) binding site (Figure 2C). Such cleavage products were not detected in RNA from wild type seedlings. Second, cleavage of *CHS *mRNA in transgenic plants should lead to a reduction in *CHS *mRNA levels as compared to wild type plants. *CHS *mRNA levels were quantified by qRT-PCR experiments using RNA extracted from transgenic lines that were identified as high pre-smiRNA(CHS) expressors. Indeed, in all investigated transgenic lines the *CHS *mRNA level was significantly reduced as compared to wild type seedlings. The degree of the down-regulation of the target mRNA was negatively correlated to the level of pre-smiRNA(CHS) over-expression (Figure 2D). Like with the over-expression of pri-smiRNA(CHS) generated by the standard protocol, very similar results were obtained in transgenic plants that over-expressed the pri-smiRNA(CHS) ECV (data not shown).

### Phenotypic analyses of transgenic lines expressing pri-smiRNA (CHS)

**Figure 3:**
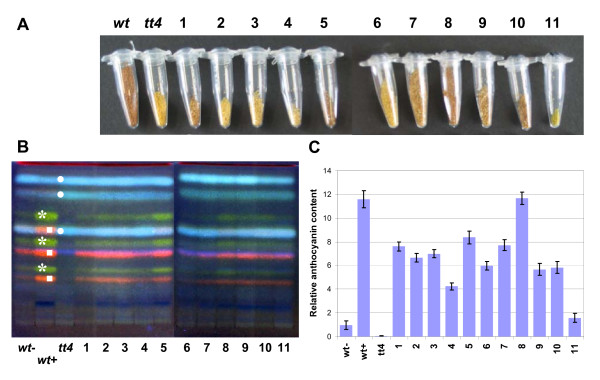
**Phenotypic analyses of transgenic lines expressing pri-smiRNA(CHS)**. **(A) **Comparison of the seed coat color of seeds from different transgenic lines numbered 1-11 expressing pri-smiRNA(CHS). Wild type (*wt*) and *chs *knockout seeds (*tt4*) are shown for reference. **(B) **Thin layer chromatography of methanolic extracts from the same transgenic lines to analyze flavonol content. Green (asterisks), kaempferol glycosides; red (squares), quercetin glycosides; blue (dots), sinapate derivatives. Wild type plants were grown either without (*wt*-) or with 4% sucrose (*wt*+). All other plants were grown with 4% sucrose. **(C) **Relative anthocyanin content in seedlings of the same transgenic lines.

In order to obtain further information on the effects of smiRNA(CHS) action in the same transgenic lines that were analyzed molecularly, we performed several experiments to detect different products or intermediates of the flavonoid biosythesis pathway. Phenotypic changes caused by the down-regulation of *CHS *mRNA, like changes in seed color, flavonol composition, and anthocyanin content were documented [26,30,31]. As an extreme example, the *chs *null mutant line *tt4 *(for *transparent testa 4*) shows yellow seed color due to the failure of cells to produce and accumulate proanthocyanidins in the seed coat. Further characteristics of *tt4 *include the lack of flavonols, like quercetin and kaempferol, as well as absence of anthocyanin accumulation under stress conditions (Figure 3).

Among 11 transgenic smiRNA(CHS) lines generated, seed coat color ranged from dark brown (similar to wild type seeds) to bright yellow (like in *tt4 *seeds), probably due to reduced proanthocyanidin content (Figure 3A). Using thin layer chromatography, methanolic extracts from transgenic seedlings were analyzed in comparison to extracts from wild type and *tt4 *plants (Figure 3B). In extracts from *tt4 *seedlings, only sinapate derivatives could be detected, but no flavonol derivatives like quercetin and kaempferol glycosides, whereas these compounds could readily be verified in samples from wild type seedlings. In all of the transgenic lines that over-expressed pri-smiRNA(CHS), quercetin and kaempferol glycosides were detected at lower concentrations, again with differences between the investigated lines (Figure 3B). Additionally, DBPA-staining was performed with whole seven-day-old transgenic seedlings to confirm the results obtained with thin layer chromatography (See additional file 3: Phenotypic analyses of transgenic lines expressing pri-smiRNA(CHS)). Yellow staining indicating the presence of flavonol derivatives was observed to a lower extent in all of the investigated smiRNA(CHS) lines than in wild type plants. Interestingly, flavonols were detected in the root tips in all of the DPBA-stained transgenic lines as well. The expression of *CHS *and hence the synthesis of anthocyanins in plants is inducible by stress, for instance by addition of sucrose to the medium and growth under high light [31]. This response was investigated in transgenic seedlings over-expressing pri-smiRNA(CHS) in comparison to wild type or *tt4 *seedlings and quantified photometrically (Figure 3C). After seven days, transgenic smiRNA(CHS) seedlings revealed lower anthocyanin content and a weaker red staining in the upper hypocotyl and in cotyledons than wild type plants growing in high light and in presence of sucrose (See additional file 3: Phenotypic analyses of transgenic lines expressing pri-smiRNA(CHS)). This analysis revealed a reduction of the anthocyanin content of up to 87% in transgenic lines as compared to wild type plants grown under identical conditions. Again, very similar results concerning seed color, flavonol composition, and anthocyanin content were obtained in transgenic plants that over-expressed the pri-smiRNA(CHS) ECV (See additional file 4: Phenotypic analyses of pri-smiRNA(CHS) ECV transgenic lines).

### Off-target analysis

**Figure 4:**
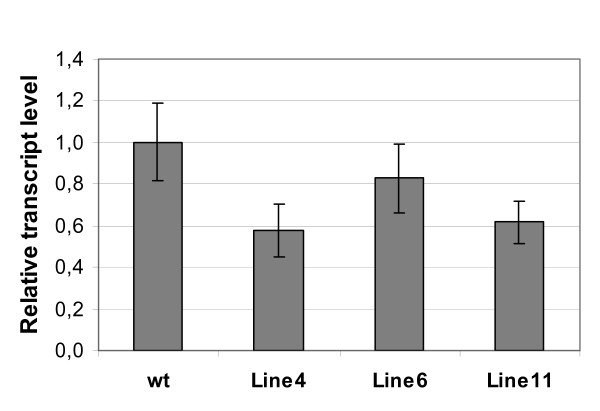
**Analysis of off-target transcript levels**. One potential off-target predicted with *RNAhybrid *was tested for smiRNA(CHS)-mediated effects on transcript abundance. RNA was extracted from different transgenic lines that highly over-expressed pri-smiRNA(CHS), and qRT-PCR experiments were performed to quantify the relative transcript levels of *At1g40390*. Data were normalized to wild type (*wt*) transcript levels. See also Figure 2B.

To minimize the occurrence of off-targets, the design of smiRNA(CHS) was aided by *in silico *hybridizations against the complete Arabidopsis transcriptome using the program *RNAhybrid*. However, one potential off-target, *At1g49390 *encoding a putative oxidoreductase of the Fe(II) oxigenase family, was found (See additional file 1: *In silico *target predictions for smiRNA(CHS) using the program *RNAhybrid*). This potential off-target was not detected using other programs that are widely used for smiRNA design [32]. In order to test whether or not *At1g49390 *is a true off-target that is detected by our prediction method, we performed qRT-PCR experiments from RNA extracted from the transgenic smiRNA(CHS) lines. Figure 4 shows that significant down-regulation of *At1g49390 *was detected in transgenic lines that over-expressed smiRNA(CHS). These results proved that *At1g49390 *is a true off-target for smiRNA(CHS), albeit its down-regulation by smiRNA(CHS) was not as strong as that measured for the main target *CHS *mRNA.

### Design of smiRNAs: mismatches and excess G:U base pairs

**Figure 5:**
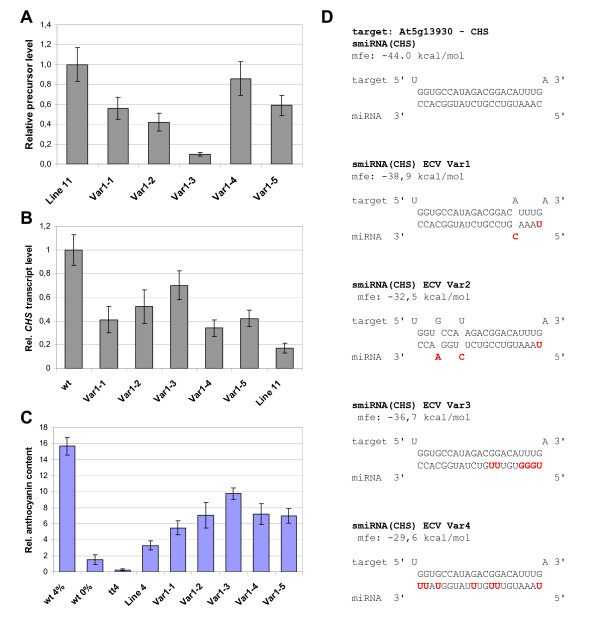
**Molecular and phenotypic analyses of transgenic lines expressing mutant variant 1 (Var1) of smiRNA(CHS) ECV**. RNA was extracted from different transgenic lines expressing smiRNA(CHS) ECV Var1, and qRT-PCR experiments were performed to quantify the relative transcript levels **(A) **of the smiRNA(CHS) ECV Var1 precursor and **(B) **of the *CHS *mRNA target. Data were normalized to the highest values. *wt*, wild type seedlings; Line 11, smiRNA(CHS) line 11 as controls (compare with Figures 2 and 3). **(C) **Relative anthocyanin content of the same transgenic smiRNA(CHS) ECV Var1 lines. Wild type seedlings were grown either without (*wt*-) or with 4% sucrose (*wt*+). All other plants were grown with 4% sucrose. Control seedlings were measured for comparison: *chs *knockout (*tt4*) and smiRNA(CHS) line 4 (Line 4; compare with Figures 2 and 3). **(D) **smiRNA:target mRNA hybrid structures of all smiRNA(CHS) variants. Differences of Var1-4 to the original smiRNA(CHS) are indicated in red. Mfe, minimal free energy.

The number of G:U base pairs and the use of mismatches in the smiRNA:mRNA hybrid structure and their effect on the level of down-regulation of the target is of critical importance for smiRNA design. This is especially true when designing smiRNAs to avoid closely related genes from being targeted or when targeting several members of multigene families. Trying to address these issues, we generated variants of the smiRNA(CHS) in the ECV backbone that (1) contained one mismatch in the 5' region or (2) two mismatches in the 3' region of the smiRNA:mRNA hybrid structure, plus a 5'U in both cases. In addition, we introduced (3) six G:U base pairs into the smiRNA:mRNA hybrid structure by exchanging Cs for Us and As for Gs in the 5' part of the smiRNA(CHS) or (4) we introduced seven G:U base pairs by exchanging all Cs for Us over the entire length of the smiRNA(CHS) (Figure 5D). The respective pri-smiRNA constructs were generated, several transgenic lines were obtained and analyzed for each smiRNA(CHS) variant. Molecular and phenotypic analyses of these transgenic lines revealed that significant down-regulation of *CHS *mRNA and the occurrence of corresponding phenotypes were observed with the smiRNA(CHS) variant containing one mismatch (Figure 5). Here again, we found negative correlation between pre-smiRNA(CHS) Var1 expression levels and *CHS *mRNA levels as well as anthocyanin content. By contrast, only very limited down-regulation of *CHS *mRNA and anthocyanin content was detected with the smiRNA(CHS) Var2 containing two mismatches. This was even more the case with smiRNA(CHS) Var3 and Var4 characterized by increased G:U base pairing to their target (See additional file 5: Molecular and phenotypic analyses of transgenic lines expressing mutant variants Var2, Var3 and Var4 of smiRNA(CHS) ECV).

## Discussion

The generation of loss-of-function lines is important for functional genome research in plants. The function of genes for which no null mutations are available must be tackled in other ways. Knockdown approaches like the constitutive or inducible expression of anti-sense RNA or RNA interference (RNAi) methods are often applied since they are technically relatively easy and fast to achieve [33]. However, the specificity of such knockdown approaches may be a problem when dealing with genes for which close homologues exist or with gene families of closely related members. The finding that the often complex secondary structure or shape of the plant precursor miRNAs is more important for processing by DCL1 than the sequence of the mature miRNA itself opened a novel door to generate highly specific loss-of-function lines in plants [21,32]. We combined the approach of generating synthetic miRNAs in plant cells with the target prediction tool *RNAhybrid *[23] to aid with the design and to minimize potential off-targets.

We designed a smiRNA to target the single gene *CHS *because loss-of-function alleles of *CHS *show easily detectable phenotypes due to the impaired flavonoid biosynthesis pathway. One group of flavonoids are the proanthocyanidins that are exclusively located in the seed coat, where they are responsible for the brown pigmentation in mature seeds that, after further oxidation processes, is believed to protect the seeds from UV-damage [34]. A *chs *null mutant, named *tt4*, has yellow seeds due to the disability of the cells to produce and accumulate proanthocyanidins in the seed coat. Further products of the flavonoid biosynthesis pathway are flavonols like quercetin and kaempferol, which can be detected by thin layer chromatography and staining methods [30]. Finally, anthocyanins accumulate in hypocotyls and cotyledons of young seedlings under stress conditions [31] and this response can be quantified easily.

We designed the first smiRNA(CHS) with no mismatches to the target (Figure 1; See additional file 1: *In silico *target predictions for smiRNA(CHS) using the program *RNAhybrid*) using the prediction program *RNAhybrid*. RNAhybrid performs *in silico *hybridizations between a miRNA and an mRNA target in a way that optimizes the free energy of the hybridization and that is consistent with user-defined constraints [23]. In addition, G:U base pairs are not treated as mismatches but contribute to a lesser extent to the overall minimal free energy of the miRNA:mRNA hybrid. The program was adapted to plant specificities and successfully used to predict many novel miRNA targets in Arabidopsis and in other plant species [27]. For smiRNA(CHS) design, we concentrated on high specificity to the target (avoidance of off-targets), on an average GC content (less than 60%), and on a reasonably low mfe value of the smiRNA:mRNA hybrid that should be equal or greater than 72% of a perfect match hybrid [21]. Usually, hybrid structures of natural miRNAs with their target mRNAs do not show more than one mismatch between the 5'end of the miRNA and the target sequence, or more than two mismatches in the 3'end. However, a perfect hybridization in the 3'end of the miRNA may compensate for mismatches in the 5'end [35]. Furthermore, natural miRNA:mRNA hybrid structures usually do not show mismatches at the site of the presumptive cleavage of the target [36]. High specificity of the designed smiRNA and these criteria formed the basis for the design and choice of smiRNA(CHS) sequence. It was reported that AGO1 preferentially incorporates small RNAs with a 5' terminal U [37]. This was taken into account when designing smiRNA(CHS) Var1-4, while the original smiRNA(CHS) was designed with a 5' terminal C. Other constraints suggested by Ossowski et al. [32] like an A or U at position 10 or a higher AU content of the smiRNA at the 5'end were not taken into consideration since they would reduce design possibilities, especially with a smiRNA that should target members of a gene family or a gene for which close homologues exist. In addition, these characteristics described by Ossowski et al. [32] are not present in all naturally occurring miRNAs.

In all transgenic plant lines containing the "standard" version of pri-smiRNA(CHS) that were analyzed we demonstrated expression of the smiRNA(CHS) and its precursor, some lines showing very high expression levels (Figure 2). In small RNA Northern blots, we detected two small RNAs hybridizing with the smiRNA(CHS) probe (Figure 2B). However, a similar situation was also observed for the endogenous miR156. In this case, small RNA Northern experiments revealed a 20 nt-long miRNA that was predicted, and an additional 21 nt-long miRNA [38]. The two prominent cleavage sites of the *CHS *mRNA target that we detected in our validation experiments (Figure 2C) may be caused by these two smiRNA(CHS) of slightly different sizes. In addition, using a qRT-PCR approach, we showed that the target mRNA levels in smiRNA(CHS) transgenic lines were significantly reduced as compared to wild type plants (Figure 2D). Interestingly, phenotypic variation (Figure 3) and down-regulation of *CHS *mRNA correlated very well with the expression level of pre-smiRNA(CHS) and with the level of smiRNA(CHS) detected on small RNA Northern blots (Figure 2). This is also obvious with seed color and with flavonol and anthocyanin content (Figure 3; See additional file 3: Phenotypic analyses of transgenic lines expressing pri-smiRNA(CHS)). The range of phenotypic variation was between being almost like in wild type plants and almost like in the *chs *knockout mutant *tt4*. Phenotypic variation of transgenic smiRNA lines was described before [21] and this phenomenon is of advantage when addressing transcripts of essential genes.

As a major simplification of the cloning procedure for pri-smiRNAs, we generated the ECV vector by introducing restriction sites flanking the original miR159a precursor to accommodate any smiRNA precursor in the pri-miR159a backbone (Figure 1). Transgenic lines expressing pri-smiRNA(CHS) from the ECV vector yielded very similar results as transgenic lines that were generated by the standard procedure (Figure 2, 3; See additional file 4: Phenotypic analyses of pri-smiRNA(CHS) ECV transgenic lines). Although we analyzed only five ECV lines, three of these lines showed yellowish seed color comparable to the *tt4 *mutant seeds or to seeds from standard line 4, a strong smiRNA(CHS) expressor. Likewise, reduction of anthocyanin content in these three ECV lines was very similar to standard line 4. Since both strategies are designed to result in the expression of the same smiRNA(CHS) these results prove that the ECV vector approach shows the same effectiveness in generating efficient knockdown lines as the standard approach.

Off-target avoidance or at least off-target minimization is very important for the specificity of smiRNA approaches. Here we showed that *RNAhybrid *is superior to other prediction methods that are widely used for smiRNA approaches in detecting true off targets [32]. Target prediction results for smiRNA(CHS) against the complete Arabidopsis transcriptome yielded only 13 candidates, using *RNAhybrid *with the specified settings (See additional file 1: *In silico *target predictions for smiRNA(CHS) using the program *RNAhybrid*). One of these 13 candidates is the target *CHS *mRNA, a second one is the off-target *At1g49390 *encoding a putative oxidoreductase. Relative transcript levels of *At1g49390 *were indeed down-regulated by smiRNA(CHS) in transgenic lines (Figure 4), but not as strongly as the *CHS *mRNA target (Figure 2). The other *RNAhybrid *hits were excluded from experimental analysis because their hybrid structures are characterized by too many bulges, loops, and/or too long unpaired overhangs to be targeted effectively by smiRNA(CHS).

The limited design of additional mismatches and/or G:U base pairs in smiRNA approaches may be a way to improve targeting specificity, especially when dealing with gene families or with genes for which close homologues exist. In an attempt to address these issues, we generated mutant variants of smiRNA(CHS) in the ECV vector backbone and analyzed the effects of their expression in transgenic lines. Variant 1 (Var1) contains one mismatch in the 5' region of the smiRNA and a 5'U, resulting in a slightly higher but still relatively low mfe value of the smiRNA:mRNA hybrid (Figure 5). Such characteristics are often found in naturally occurring miRNA:mRNA hybrids as well. Accordingly, seed color was yellowish in most transgenic lines (data not shown), and the reduction of *CHS *transcript levels in some transgenic lines almost reached that of the strong smiRNA(CHS) expressor line 11 (Figure 5). Again, the expression level of the pre-smiRNA(CHS) Var1 was negatively correlated to target *CHS *mRNA levels, very similar to the situation found with smiRNA(CHS) carrying no mismatches (Figure 2, 3). The same was true for the reduction of the anthocyanin level in the transgenic smiRNA(CHS) Var1 seedlings as compared to the strong smiRNA(CHS) expressor line 4 (Figure 5). The fact that the levels of target mRNA and anthocyanin reduction of the reference lines were only almost reached with transgenic smiRNA(CHS) Var1 lines could be due to the relative small number of transgenic smiRNA(CHS) Var1 lines that were recovered and analyzed. Although it is very difficult to quantify the effect of variations in smiRNA sequence due to very different expression levels in the transgenic lines, the conclusion could be drawn that smiRNA(CHS) Var1 was also very effective in knocking down *CHS *expression and in producing related phenotypes.

The introduction of two mismatches in the 3' region and a 5'U in smiRNA(CHS) Var2 also leads to a higher but still relatively low mfe value of the smiRNA:mRNA hybrid that is 73.8% of the mfe value of a perfect match hybrid (Figure 5D). In this case, there was only limited correlation of pre-smiRNA(CHS) Var2 expression levels and the degree of anthocyanin reduction in the transgenic lines analyzed (See additional file 5: Molecular and phenotypic analyses of transgenic lines expressing mutant variants Var2, Var3 and Var4 of smiRNA(CHS) ECV). However, we detected a slight reduction of anthocyanin levels as compared to the wild type, but by far not as strong as in the reference line 4. Although complementarity of the 5' region of the miRNA to its target is thought to be more important for miRNA function, two mismatches in the 3' region of miRNA:mRNA hybrids are rare in naturally occurring miRNAs. As a conclusion, two loops in the 3' region of the smiRNA, each with one mismatched nucleotide on either side, greatly reduce knockdown efficiency.

Knockdown efficiencies were even more reduced in smiRNA(CHS) Var3 and Var4 lines that are characterized by increased G:U base pairing with their target. The smiRNAs, however, were detected in transgenic lines containing smiRNA(CHS) variants Var1-4, as shown by small RNA Northern blots (See additional file 5: Molecular and phenotypic analyses of transgenic lines expressing mutant variants Var2, Var3 and Var4 of smiRNA(CHS) ECV). smiRNA(CHS) Var3 and Var4 did not contain mismatches to the *CHS *mRNA target. However, the sequence changes led to increased mfe values of the respective hybrid structures and to an increase in the degrees of freedom with respect to hybridization partners (Figure 5D). The mfe values of the smiRNA:mRNA hybrids of smiRNA(CHS) Var3 and Var4 are 83.4% and 67.3% of the mfe value of a perfect match hybrid, respectively. We measured only very limited correlation of pre-smiRNA(CHS) Var3 and Var4 expression levels and the degree of anthocyanin reduction in the transgenic lines analyzed (See additional file 5: Molecular and phenotypic analyses of transgenic lines expressing mutant variants Var2, Var3 and Var4 of smiRNA(CHS) ECV). Again, it is very difficult to quantify the effect of variations in smiRNA sequence due to very different expression levels in the transgenic lines and due to the limited number of transgenic lines analyzed. However, there was the clear tendency that excess G:U base pairs in either the 5' region of the smiRNA or over its entire length strongly decreased knockdown efficiency.

## Conclusion

We improved the design and generation of pri-smiRNAs by (1) the use of the target prediction program *RNAhybrid *for sensitive detection and thus effective avoidance of off-targets during smiRNA design, and by (2) the generation of an "easy cloning vector" (ECV) that allows a one-step cloning procedure of a double stranded DNA fragment of any smiRNA precursor into restriction sites within the cDNA of the *MIR159A *primary transcript, thereby replacing the original miR159a and miR159a*. We demonstrated that the "ECV" approach was as effective to generate efficient knockdown lines as was the "standard" procedure. As proof-of-principle, we down-regulated the relative expression level of *CHS *mRNA in the strongest smiRNA(CHS) expressing line to 15% of its level in wild type seedlings, corresponding to a reduction of the anthocyanin content to 13% of the level found in wild type seedlings grown under identical conditions. For strong knockdown efficiencies, sequence variations of the smiRNA vs. the mRNA target should be limited to one mismatch in the 5' or 3' region and the use of G:U base pairs should be restricted as well.

## Methods

### Design of synthetic microRNAs

The first step consisted of a BLASTN search [39] with the target cDNA to identify close homologues. Then, the alignment tool Clustal W [40] was used to help select sequence regions within candidate target cDNAs that show highest specificity for the chosen target. Candidate smiRNA sequences were selected on the basis that there should be no mismatches between the smiRNA and the mRNA target at the presumed cleavage site around nucleotides 10 and 11 of the smiRNA, and bulges and loops should be limited to one and two nucleotides, respectively, or even be avoided. The 5'end of the smiRNA should show near-perfect complementarity. As next steps, the program *RNAhybrid *http://bibiserv.techfak.uni-bielefeld.de/rnahybrid/ was used to calculate minimal free energy (mfe) values for the candidate smiRNA:mRNA hybrid structures that should be -25 kcal/mol or below for optimal hybridization and to search for potential off-targets within the complete Arabidopsis transcriptome. Settings were: maximum internal loop size: 2 nt; maximum bulge size: 1 nt; minimal free energy cutoff: -25 kcal/mol; p-value cutoff: 0,001. Depending on the outcome of these tests the candidate smiRNA sequence was adjusted accordingly and the calculations were repeated (See additional file 1: *In silico *target predictions for smiRNA(CHS) using the program *RNAhybrid*).

Once the smiRNA sequence was chosen, the sequence of the corresponding smiRNA* was designed in a way that preserved the mfe value and the loop of the original *MIR159A *backbone within the smiRNA:smiRNA* hybrid structure. Arabidopsis *MIR159A *was amplified by PCR from genomic *Arabidopsis thaliana Col-0 *DNA and inserted into the *XbaI *and blunted *SacI *restriction sites of pUC19-3'GFP (Figure 1) [27,41], thereby exchanging the GFP cDNA for pri-miR159a (Primers are given in additional file 6: Oligo nucleotide sequences). This *MIR159A *sequence, termed pri-miR159a in this work, is much longer than the miR159a precursor, however, it does not contain the transcription start site published by Xie et al. [14]. *RNAfold *[29] was then used to check the secondary structure prediction of the resulting entire pri-smiRNA, and the smiRNA* sequence was adjusted and *RNAfold *analysis repeated if necessary. According to our *in silico *predictions, however, the pre-miR159a forms a very stable stem-loop structure within pri-miR159a that seems to tolerate different smiRNA:smiRNA* sequence variations without any effect on secondary structure (See additional file 2: Sequences and predicted secondary structures of pri-smiRNAs using the program *RNAfold*). Finally, primers were designed for overlap-extension PCR [28] to generate and clone the pri-smiRNA within the endogenous *MIR159A *primary transcript as backbone [14] using the "standard" procedure (Figure 1A, 1B). Alternatively, primers containing restriction sites (*NheI *- smiRNA* sequence and *BsrGI *- smiRNA sequence) were designed for the "ECV" procedure (Figure 1C; Primer sequences are given in additional file 6: Oligo nucleotide sequences). The ECV plasmid is available upon request. The expression cassettes, including the 35S promoter and the nopaline synthase terminator, flanked by the *HindIII *and *EcoRI *restriction sites were then excised and ligated into the *HindIII *and *EcoRI *sites of the binary vector pGPTV-BAR [42].

### Plant material and growth conditions

*Arabidopsis thaliana *plants (ecotype Col-0) were cultivated on soil in a greenhouse at 24°C. They were first grown under short day conditions (8 h light/16 h darkness) for 2-3 weeks and then transferred to long day conditions (16 h light/8 h darkness) until seeds were harvested. Alternatively, surface-sterilized *Arabidopsis thaliana *seeds were sown on 0.8% agar plates containing 0.5 × Murashige-Skoog basal salts (Duchefa, Haarlem, Netherlands), with (4%) or without sucrose, kept at 4°C in the dark for 3 days for stratification, and then transferred to a light chamber with 16 h of light per day at 22°C until the plant material was harvested for further analysis.

### Agrobacterium-mediated transformation of *Arabidopsis thaliana*

*Agrobacterium tumefaciens *(GV3101) was transformed with the binary vector pGPTV-BAR carrying the pri-smiRNAs under the control of the 35S cauliflower mosaic virus promoter. A small scale Agrobacteriumpre-culture was grown for two days at 28°C at 200 rpm in 5 ml YEP medium (1% DIFCO Bacto Tryptone (w/v), 1% DIFCO Bacto Yeast Extract (w/v), 0.5% NaCl (w/v)) containing appropriate antibiotics. This was used to inoculate a large scale culture of 500 ml YEP that was grown for two days at 28°C at 200 rpm. Sucrose (5%) and Silwet L-77 (0,02%, Lehle Seeds, USA) were added to the culture that was incubated for another 10 min at 28°C. Plants were dipped upside down in the bacterial solution for 2 min, drained, placed on the side, covered for 24 hours in Saran wrap, and then grown until seed harvest. Seeds of the T1 generation were harvested, T1 transgenic plants were selected by BASTA treatment, and the survivors were grown to harvest T2 seeds.

### Small RNA Northern blots

Total RNA from *Arabidopsis thaliana *plants was isolated using TRI reagent (Molecular Research Center, Cincinnati, USA) and separated on a 17% denaturing polyacrylamide gel containing 7 M urea in TBE buffer (0.9 M Tris, 0.9 M boric acid, 0.02 M EDTA). The RNA was electro-blotted to Hybond N^+ ^nylon membranes (GE Healthcare, UK) for 1 h at 400 mA using a trans-blot transfer cell (Bio-Rad, Hercules, CA, USA) and crosslinked by UV light (StrataLinker 1800, Stratagene, La Jolla, CA, USA). Pre-hybridization and hybridization of the blots with biotinylated single-stranded DNA probes (oligonucleotides that contained the reverse-complementary sequence of the smiRNA) were carried out in PerfectHyb Plus Hybridization Buffer (Sigma-Aldrich Co., St. Louis, MO, USA) at 42°C for 1 h and overnight, respectively. Blots were washed with decreasing concentrations of SSC/SDS (2 × SSC, 0.2% SDS; 1× SSC, 0.1% SDS; 0.5× SSC, 0.1% SDS) at 50°C. Detection was carried out with the Chemiluminescent Nucleic Acid Detection Module (Pierce, Rockford, IL, USA) according to the manufacturers' instructions, and membranes were exposed to BioMax XAR Films (Kodak, USA). Blots were stripped in between hybridizations by 10 min incubation with 1% SDS at 80°C. The second hybridization was with the U6 snRNA probe to detect U6 snRNA as loading control and a 21 nt-long RNA size marker.

### Analysis of secondary metabolites in *Arabidopsis thaliana*

In order to analyze the content of flavonol derivatives in transgenic *Arabidopsis thaliana *plants, methanolic extracts were isolated from 7-days-old whole seedlings using 80% methanol and 10-15 zirconia beads of 1 mm diameter (Roth, Karlsruhe, Germany). Homogenized samples (Tissue Lyser, Qiagen, Retsch, Germany) were incubated for 10 min at 65°C and centrifuged for 10 min at 4°C at 14000 rpm in a standard table centrifuge. Supernatants were vacuum-dried in a SpeedVac (SPD111-V, Thermo Electron, Waltham, MA, USA) at 60°C. Dried pellets were dissolved in 1 μl of 80% methanol per mg fresh weight starting material. From each sample, 4 μl were used for high-performance thin layer chromatography [30] and spotted on 10 cm × 10 cm silica-60 HPTLC-plates (Merck, Darmstadt, Germany) as the stationary phase. The chromatography was carried out using ethyl acetate, formic acid, acetic acid and water (100:26:12:12) as the mobile phase in a closed glass tank. Separated compounds were stained by spraying a 1% (w/v) DPBA solution (Diphenyl boric acid-β-aminoethylester, Naturstoffreagenz A, Roth, Karlsruhe, Germany) [30] in methanol, followed by spraying 5% (w/v) methanolic polyethylene glycol 4000 solution. The stained HPTLC plates were examined under UV light (312 nm) and photographed.

For the visualization of flavonols in whole Arabidopsis seedlings, seeds were germinated and grown for 5 days on filter paper soaked with 3 ppm norflurazon in water (Sigma-Aldrich, Co., St. Louis, MO, USA) under long-day conditions. Bleached seedlings were stained to saturation for at least 1.5 h in a freshly prepared solution of 0.25% (w/v) DPBA in 0.00375% (v/v) Triton X-100. Fluorescence was visualized with a Leica DM5500 B epifluorescence microscope using Leica Filtercube A with an excitation wavelength of 340-380 nm and a 425-nm-long-pass splitter.

For the quantification of the accumulation of anthocyanins, transgenic plants were grown on 0.5 × MS medium enriched with 4% sucrose under long-day conditions to induce the formation of stress anthocyanins. Anthocyanins were extracted from whole 7 day-old seedlings by overnight incubation in acidic methanol (1% v/v HCl 37%, 99% v/v methanol p.a.) with gentle shaking. Samples were centrifuged for 1 min at 14000 rpm and room temperature in a standard table centrifuge and 0.5 ml of the supernatants were used to measure the absorption at 530 nm and 657 nm in triplicates. The relative quantity of anthocyanins per g fresh weight (FW) was calculated according to the equation Q(Ant) = (OD(530)-0,25 × OD(657))/FW [g^-1^].

### Quantitative RT-PCR experiments

Primers for quantitative RT-PCR (qPCR) were designed to flank the predicted smiRNA binding site in the *CHS *(target) or *At1g49390 *(off-target) transcripts, respectively, and tested with BLASTN [38] against the Arabidopsis transcriptome to ensure specificity (Primer sequences are given in additional file 6: Oligo nucleotide sequences). Reverse transcription reactions were carried out in 20 μl volume using 4 μg DNaseI-treated total RNA (DNA-free kit, Ambion, Austin, TX, USA) and Superscript II reverse transcriptase (Invitrogen, Karlsruhe, Germany) according to the manufacturers' instructions. qPCR reactions were performed using the Platinum SYBR Green qPCR SuperMix-UDG kit (Invitrogen, Karlsruhe, Germany) on a Rotor Gene 6000 Cycler (Corbett Research, Mortlake, NSW, Australia). Data was analyzed according to Pfaffl [43] and average results of two biological replicates, each with triple samples, are given.

### Target Validation experiments

RNA ligase-mediated rapid amplification of 5'cDNA ends (RLM-5'RACE) was performed with RNA that was isolated with TRI reagent (Molecular Research Center, Cincinnati, USA) from *Arabidopsis thaliana *plants. An RNA primer was directly ligated to total RNA from whole seedlings using T4 RNA Ligase (New England Biolabs, Ipswich, England), and adaptor-ligated total RNA was used for reverse transcription with SuperScript II reverse transcriptase (Invitrogen, Karslruhe, Germany) using oligo dT primers. The cDNA was subjected to nested PCR using 5' adaptor-specific primers and 3' gene-specific primers. PCR products were gel-purified, cloned into TOPO TA vector (pCR2.1; Invitrogen, Karlsruhe, Germany) and then sequenced (Primer sequences are given in additional file 6: Oligo nucleotide sequences).

## Competing interests

The authors declare that they have no competing interests.

## Authors' contributions

SN performed most of the experiments and bioinformatic analyses, LAJ established the 5'RACE experiments, helped with bioinformatics and performed part of the cloning experiments, TM designed the experiments, and SN and TM prepared the manuscript that all authors read and approved.

## Supplementary Material

Additional file 1 ***In silico *target predictions for smiRNA(CHS) using the program *RNAhybrid***. The complete Arabidopsis transcriptome dataset was downloaded from TAIR and used for *in silico *target prediction with smiRNA(CHS). The settings were: maximum internal loop size: 2 nt on either strand; maximum bulge size: 1 nt; minimal free energy cutoff: -25 kcal/mol; p-value cutoff: 0,001. The *CHS *gene (*At5g13930*) and a potential off-target (*At1g49390*) that were subjected to further analysis in transgenic smiRNA(CHS) lines are highlighted. Click here for file

Additional file 2 **Sequences and predicted secondary structures of pri-smiRNAs using the program *RNAfold***. **(A) **Endogenous pri-miR159a; **(B) **pri-smiRNA(CHS); **(C) **pri-smiRNA(CHS) ECV, the blue arrow indicates the small extra loop that is due to the introduction of restriction sites. The inserts show a magnification of the structures, highlighting the miRNA:miRNA* or smiRNA:smiRNA* hybrid, respectively. The miRNA or smiRNA is indicated with a red line, the precursor is indicated by an orange box in (A). **(D) **DNA sequences of pri-miR159a and pri-miR159a-ECV. Click here for file

Additional file 3 **Phenotypic analyses of transgenic lines expressing pri-smiRNA(CHS)**. **(A) **Close-up of seeds from different lines. **(B) **DPBA staining of whole seedlings of different lines to indicate flavonol glycosides (compare with Figure 3B). **(C) **Documentation of anthocyanin accumulation in whole seedlings, as obvious in wild type seedlings grown on 4% sucrose (*wt *4%) to induce stress anthocyanins, indicated by arrows pointing to hypocotyl and to cotyledon margins. *wt *0%, wild type seedlings grown in the absence of sucrose; *tt4*, *chs *knockout line. Click here for file

Additional file 4 **Phenotypic analyses of pri-smiRNA(CHS) ECV transgenic lines**. **(A) **Seed coat color of seeds from different smiRNA(CHS) ECV lines in comparison to standard smiRNA(CHS) line 4, wild type (*wt*) and *chs *knockout (*tt4*) seeds. **(B) **Relative anthocyanin content of the same transgenic smiRNA(CHS) ECV lines in comparison to standard smiRNA(CHS) line 4, wild type grown on 4% (*wt *4%) or without (*wt *0%) sucrose. All other plants were grown with 4% sucrose. *chs *knockout (*tt4*) seedlings were measured for comparison. Click here for file

Additional file 5 **Molecular and phenotypic analyses of transgenic lines expressing mutant variants Var2, Var3 and Var4 of smiRNA(CHS) ECV**. RNA was extracted from different transgenic lines, and qRT-PCR experiments were performed to quantify the relative transcript levels **(A, C, E) **of the smiRNA(CHS) ECV Var2, Var3 and Var4 precursors. smiRNA(CHS) line 11 (Line 11; see Figures 2 and 3) was used for normalization. **(B, D, F) **Relative anthocyanin content of the same transgenic lines. Wild type seedlings were grown either without (*wt *0%) or with 4% sucrose (*wt *4%). All other plants were grown with 4% sucrose. *chs *knockout (*tt4*) and smiRNA(CHS) line 4 (Line 4; see Figures 2 and 3) seedlings were measured for comparison. **(G) **RNA was extracted from transgenic lines expressing pri-smiRNA(CHS) Var1-4 and used for small RNA Northern blots to detect smiRNA production. RNA from wild type (*wt*) plants served as control. The upper panel shows signals obtained with the smiRNA probe, the signals in the lower panel were obtained using a probe for U6snRNA as loading control. Size marker (M): 21 nt-long RNA oligonucleotide. Click here for file

Additional file 6 Oligo nucleotide sequences. Click here for file
