# Supplementary material for: Improvement of the design and generation of highly specific plant knockdown lines using primary synthetic microRNAs (pri-smiRNAs)
Source: BMC Res Notes. 2010 Mar 4;3:59. doi: 10.1186/1756-0500-3-59 (PMC2845148; doi:10.1186/1756-0500-3-59)
Supplement: Additional file 1 — In silico target predictions for smiRNA(CHS) using the program RNAhybrid. The complete Arabidopsis transcriptome dataset was downloaded from TAIR and used for in silico target prediction with smiRNA(CHS). The settings were: maximum internal loop size: 2 nt on either strand; maximum bulge size: 1 nt; minimal free energy cutoff: -25 kcal/mol; p-value cutoff: 0,001. The CHS gene (At5g13930) and a potential off-target (At1g49390) that were subjected to further analysis in transgenic smiRNA(CHS) lines are highlighted. [file 1756-0500-3-59-S1.PDF]

```
target: At1g49390.1
length: 1047
miRNA : smiRNA_CHS
length: 21

mfe: -32.1 kcal/mol
p-value: 0.000944

position  116
target 5' C       G           C  3'
           GGUGCCG AGAUGGAUAU
           CCACGGU UCUGCCUGUAA
miRNA  3'         A           AC 5'
```

```
target: At1g52905.1
length: 144
miRNA : smiRNA_CHS
length: 21

mfe: -26.4 kcal/mol
p-value: 0.000612

position  108
target 5'   C          A   CG C  3'
             GCCGUGGACG GGC  U
             CGGUAUCUGC CUG  A
miRNA  3' CCA              UA AC 5'
```

```
target: At1g53625.1
length: 270
miRNA : smiRNA_CHS
length: 21

mfe: -27.8 kcal/mol
p-value: 0.000915

position  198
target 5' U    G  G      UG  GG  G 3'
           GGUG CG AGACGG  GU  UG
           CCAC GU UCUGCC  UA  AC
miRNA  3'      G  A      UG  A     5'
```

```
target: At2g35460.1
length: 717
miRNA : smiRNA_CHS
length: 21

mfe: -30.9 kcal/mol
p-value: 0.000936

position  501
target 5' C      G         G G    3'
           GGUGCC GUGGACGGA A
           CCACGG UAUCUGCCU U
miRNA  3'                  G AAAC 5'
```

```
target: At2g47760.1
length: 1317
miRNA : smiRNA_CHS
length: 21

mfe: -33.5 kcal/mol
p-value: 0.000674

position  407
target 5' U          UG       A  3'
           GGUGCCAUGG  GG CAUU
           CCACGGUAUC  CC GUAA
miRNA  3'            UG  U    AC 5'
```

```
target: At3g04230.1
length: 441
miRNA : smiRNA_CHS
length: 21

mfe: -30.2 kcal/mol
p-value: 0.000574

position  211
target 5' C      C      UG  A   3'
           GUGCCA GGGCGG  GU
           CACGGU UCUGCC  UA
miRNA  3' C      A      UG  AAC 5'
```

```
target: At3g16230.1
length: 1350
miRNA : smiRNA_CHS
length: 21

mfe: -33.7 kcal/mol
p-value: 0.000635

position  115
target 5' A                 A   3'
           GUGCCAUGGAUGGAUAU
           CACGGUAUCUGCCUGUA
miRNA  3' C                 AAC 5'
```

```
target: At4g15955.1
length: 537
miRNA : smiRNA_CHS
length: 21

mfe: -31.2 kcal/mol
p-value: 0.000473

position  317
target 5' A    U      UG      G   3'
           GGUG UCGUAG  GGACAU
           CCAC GGUAUC  CCUGUA
miRNA  3'             UG      AAC 5'
```

```
target: At5g01300.1
length: 489
miRNA : smiRNA_CHS
length: 21

mfe: -31.7 kcal/mol
p-value: 0.000300

position  197
target 5' U                  G   3'
           GGUGCCAUGGACGG UGU
           CCACGGUAUCUGCC GUA
miRNA  3'              U   AAC 5'
```

```
target: At5g02730.1
length: 618
miRNA : smiRNA_CHS
length: 21

mfe: -30.4 kcal/mol
p-value: 0.000948

position  432
target 5' U         U    U    A  3'
           GGUGCCAUG GCGG CAUU
           CCACGGUAU UGCC GUAA
miRNA  3'           C    U    AC 5'
```

```
target: At5g03350.1
length: 825
miRNA : smiRNA_CHS
length: 21

mfe: -31.4 kcal/mol
p-value: 0.000912

position  750
target 5' U           C  U      G  3'
           GGUGCCAUGGG CG GGCGUU
           CCACGGUAUCU GC CUGUAA
miRNA  3'                       AC 5'
```

```
target: At5g13930.1
length: 1188
miRNA : smiRNA_CHS
length: 21

mfe: -44.0 kcal/mol
p-value: 0.000003

position  771
target 5' U                     A 3'
           GGUGCCAUAGACGGACAUUUG
           CCACGGUAUCUGCCUGUAAAC
miRNA  3'                         5'
```

```
target: At5g53720.1
length: 303
miRNA : smiRNA_CHS
length: 21

mfe: -28.2 kcal/mol
p-value: 0.000900

position  217
target 5'    A             A    3'
              CCAUAGACGGACG
              GGUAUCUGCCUGU
miRNA  3' CCAC             AAAC 5'
```
