# Supplementary material for: Improvement of the design and generation of highly specific plant knockdown lines using primary synthetic microRNAs (pri-smiRNAs)
Source: BMC Res Notes. 2010 Mar 4;3:59. doi: 10.1186/1756-0500-3-59 (PMC2845148; doi:10.1186/1756-0500-3-59)
Supplement: Additional file 2 — Sequences and predicted secondary structures of pri-smiRNAs using the program RNAfold. (A) Endogenous pri-miR159a; (B) pri-smiRNA(CHS); (C) pri-smiRNA(CHS) ECV, the blue arrow indicates the small extra loop that is due to the introduction of restriction sites. The inserts show a magnification of the structures, highlighting the miRNA:miRNA* or smiRNA:smiRNA* hybrid, respectively. The miRNA or smiRNA is indicated with a red line, the precursor is indicated by an orange box in (A). (D) DNA sequences of pri-miR159a and pri-miR159a-ECV. [file 1756-0500-3-59-S2.PDF]

A
pri-miR159a

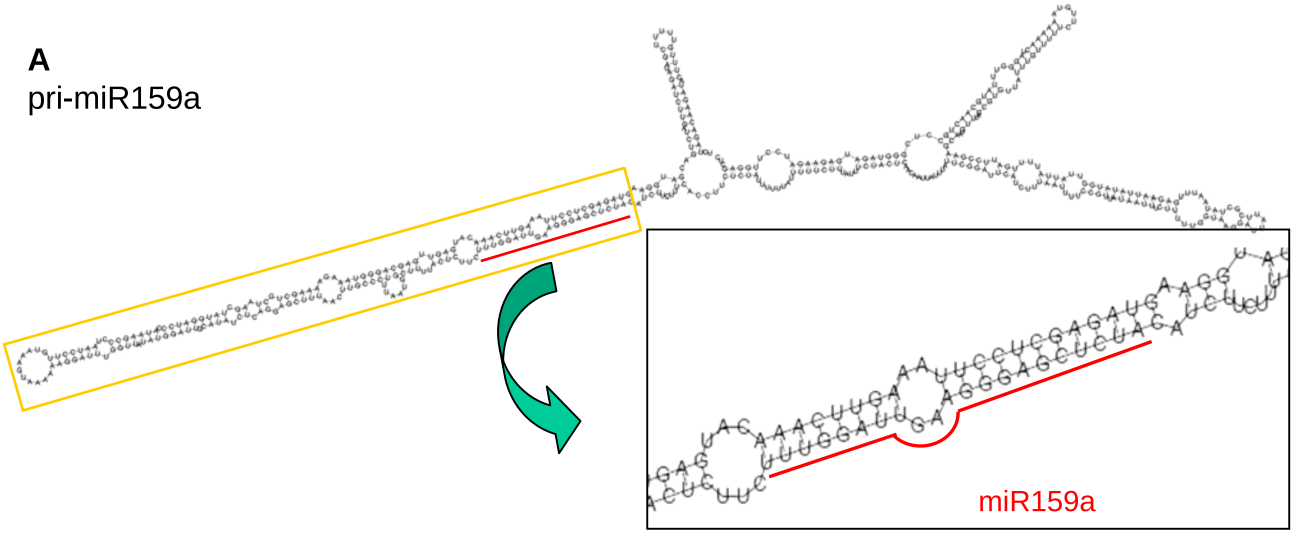

B
pri-miRNA(CHS)

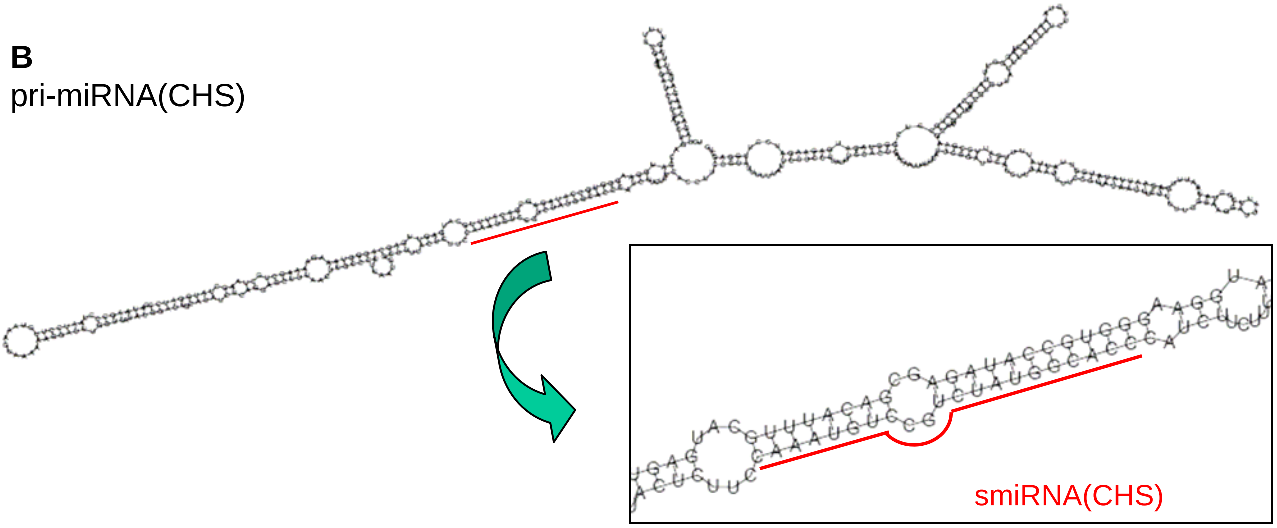

C
pri-miRNA(CHS) ECV

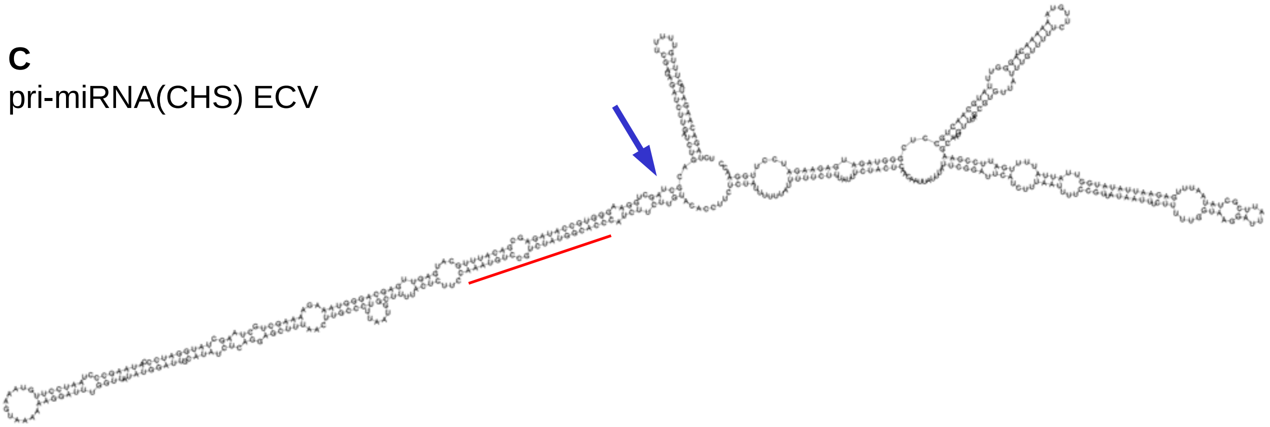

D Primary miRNA sequences

pri-miR159a: genomic Arabidopsis sequence containing the restriction sites XbaI at the 5' end and SacI at the 3' end (red) that were added for cloning.

pri-miR159a-ECV: modified genomic Arabidopsis sequence that contains, in addition to the restriction sites XbaI and SacI, the restriction sites NheI and BsrGI (blue) that were introduced for easy cloning of the smiRNA and smiRNA* sequences.

AT1G73687: DNA sequence of the precursor miR159a (miR159a DNA sequence in green).

miR159a: mature miR159a.

```
>pri-miR159a
tctagacaagatactttgtttttcgatagatcttgatctgacgatggaagtagagctccttaaagttcaaacatg
agttgagcagggtaaagaaaagctgctaagctatggatcccataagccctaatccttgtaaagtaaaaaaggatt
tggttatatggattgcatatctcaggagctttaacttgccctttaatggcttttactcttctttggattgaaggg
agctctacatcttctttcaccttctctatttttattttttctttatttctactcaacaattatttattcggattc
atctttaattttccgttataatttctttttggtaaggattattcgctataatttgagaattatatggttattatt
ttgattccgaagcaatgtttagcgtgttatttgtttttcttgtaaaaaactagggtttatgcaactgcctcgggt
agatgagaagatccttggagctc

>pri-miR159a-ECV
tctagacaagatactttgtttttcgatagatcttgatctgacgctagctggaagtagagctccttaaagttcaaa
catgagttgagcagggtaaagaaaagctgctaagctatggatcccataagccctaatccttgtaaagtaaaaaag
gatttggttatatggattgcatatctcaggagctttaacttgccctttaatggcttttactcttctttggattga
agggagctctacatcttcttgtacaccttctctatttttattttttctttatttctactcaacaattatttattc
ggattcatctttaattttccgttataatttctttttggtaaggattattcgctataatttgagaattatatggtt
attattttgattccgaagcaatgtttagcgtgttatttgtttttcttgtaaaaaactagggtttatgcaactgcc
tcgggtagatgagaagatccttggagctc

>AT1G73687
tagagctccttaaagttcaaacatgagttgagcagggtaaagaaaagctgctaagctatggatcccataagccct
aatccttgtaaagtaaaaaaggatttggttatatggattgcatatctcaggagctttaacttgccctttaatggc
ttttactcttctttggattgaagggagctcta

>miR159a
uuuggauugaagggagcucua
```
