# Supplementary figures and images for: Improvement of the design and generation of highly specific plant knockdown lines using primary synthetic microRNAs (pri-smiRNAs)
Source: BMC Res Notes. 2010 Mar 4;3:59. doi: 10.1186/1756-0500-3-59 (PMC2845148; doi:10.1186/1756-0500-3-59)

**A**

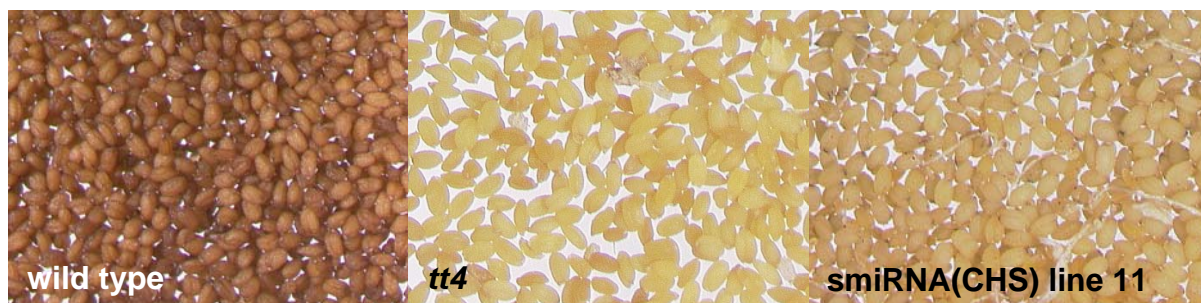

**B**

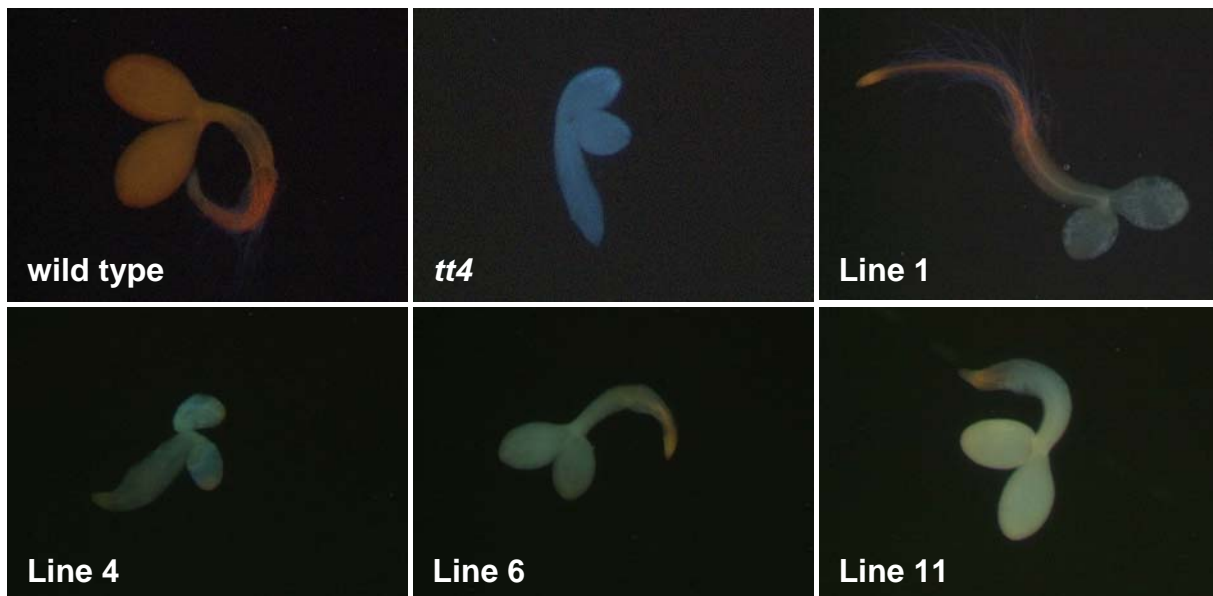

**C**

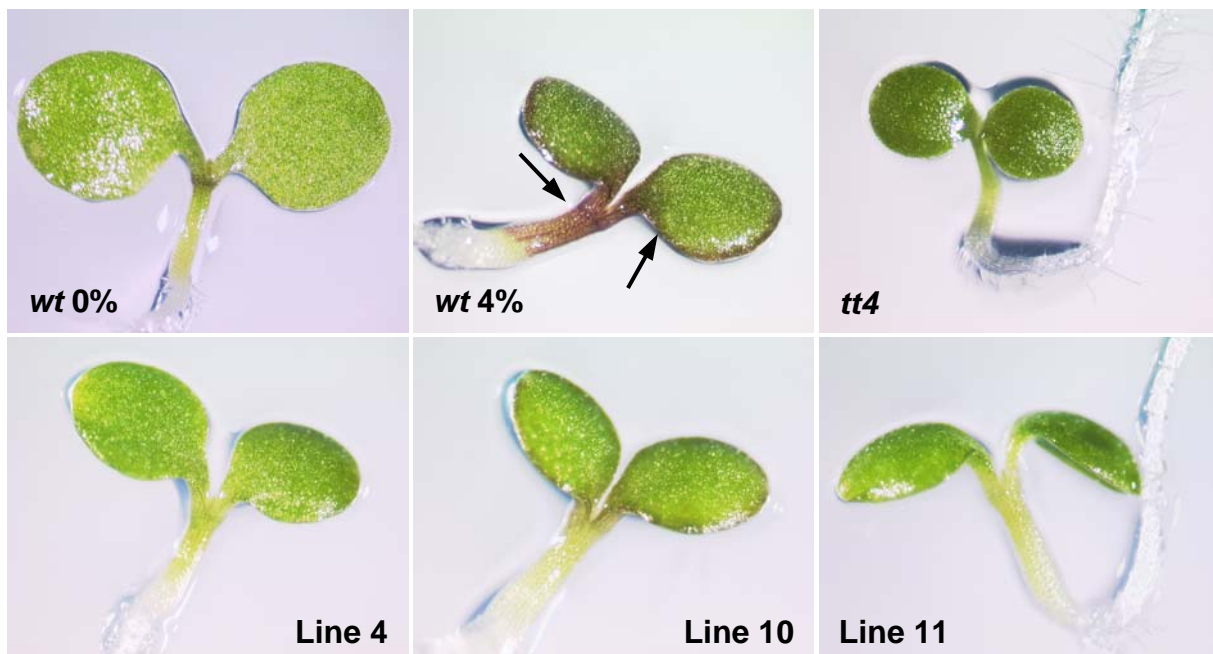

Supplement: Additional file 3 — Phenotypic analyses of transgenic lines expressing pri-smiRNA(CHS). (A) Close-up of seeds from different lines. (B) DPBA staining of whole seedlings of different lines to indicate flavonol glycosides (compare with Figure 3B). (C) Documentation of anthocyanin accumulation in whole seedlings, as obvious in wild type seedlings grown on 4% sucrose (wt 4%) to induce stress anthocyanins, indicated by arrows pointing to hypocotyl and to cotyledon margins. wt 0%, wild type seedlings grown in the absence of sucrose; tt4, chs knockout line. [file 1756-0500-3-59-S3.PDF]

**A**

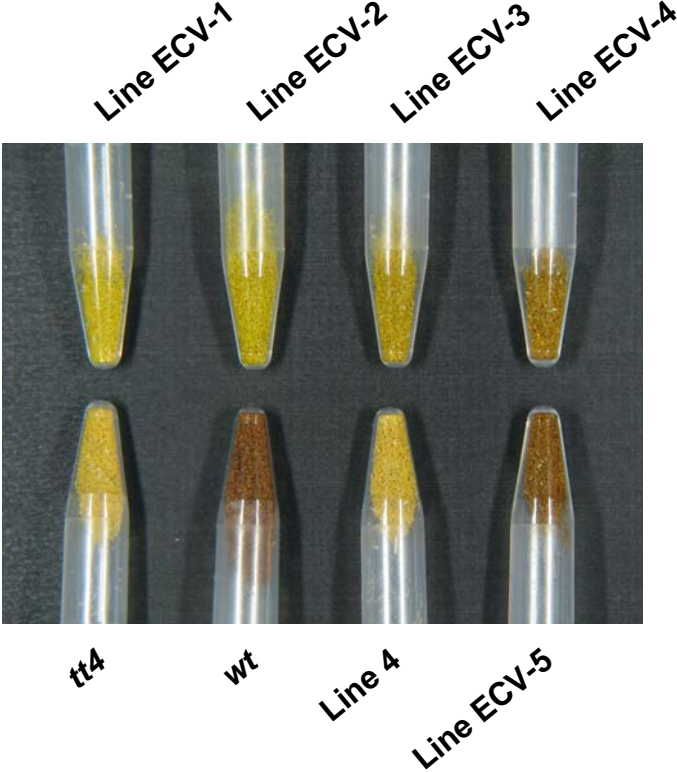

**B**

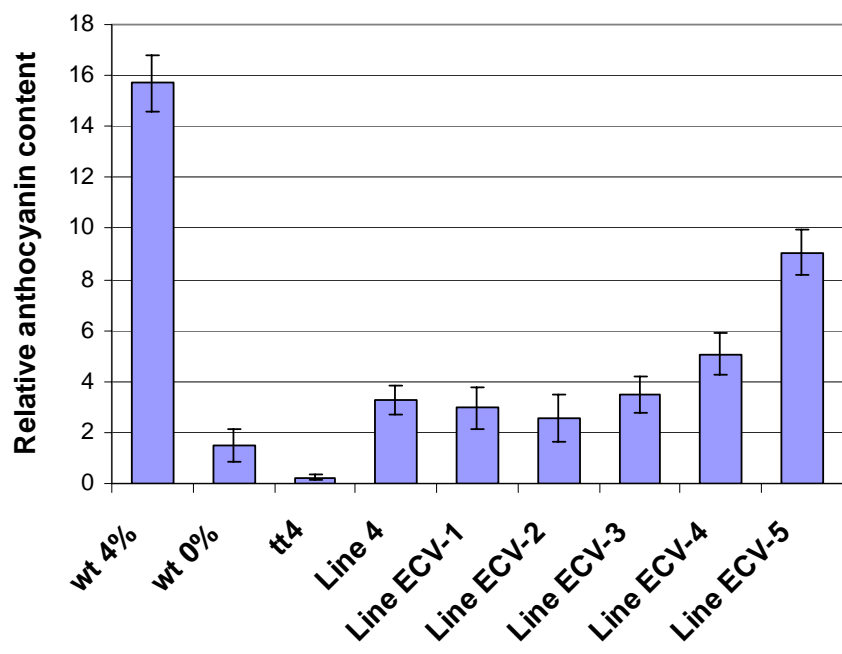

Supplement: Additional file 4 — Phenotypic analyses of pri-smiRNA(CHS) ECV transgenic lines. (A) Seed coat color of seeds from different smiRNA(CHS) ECV lines in comparison to standard smiRNA(CHS) line 4, wild type (wt) and chs knockout (tt4) seeds. (B) Relative anthocyanin content of the same transgenic smiRNA(CHS) ECV lines in comparison to standard smiRNA(CHS) line 4, wild type grown on 4% (wt 4%) or without (wt 0%) sucrose. All other plants were grown with 4% sucrose. chs knockout (tt4) seedlings were measured for comparison. [file 1756-0500-3-59-S4.PDF]

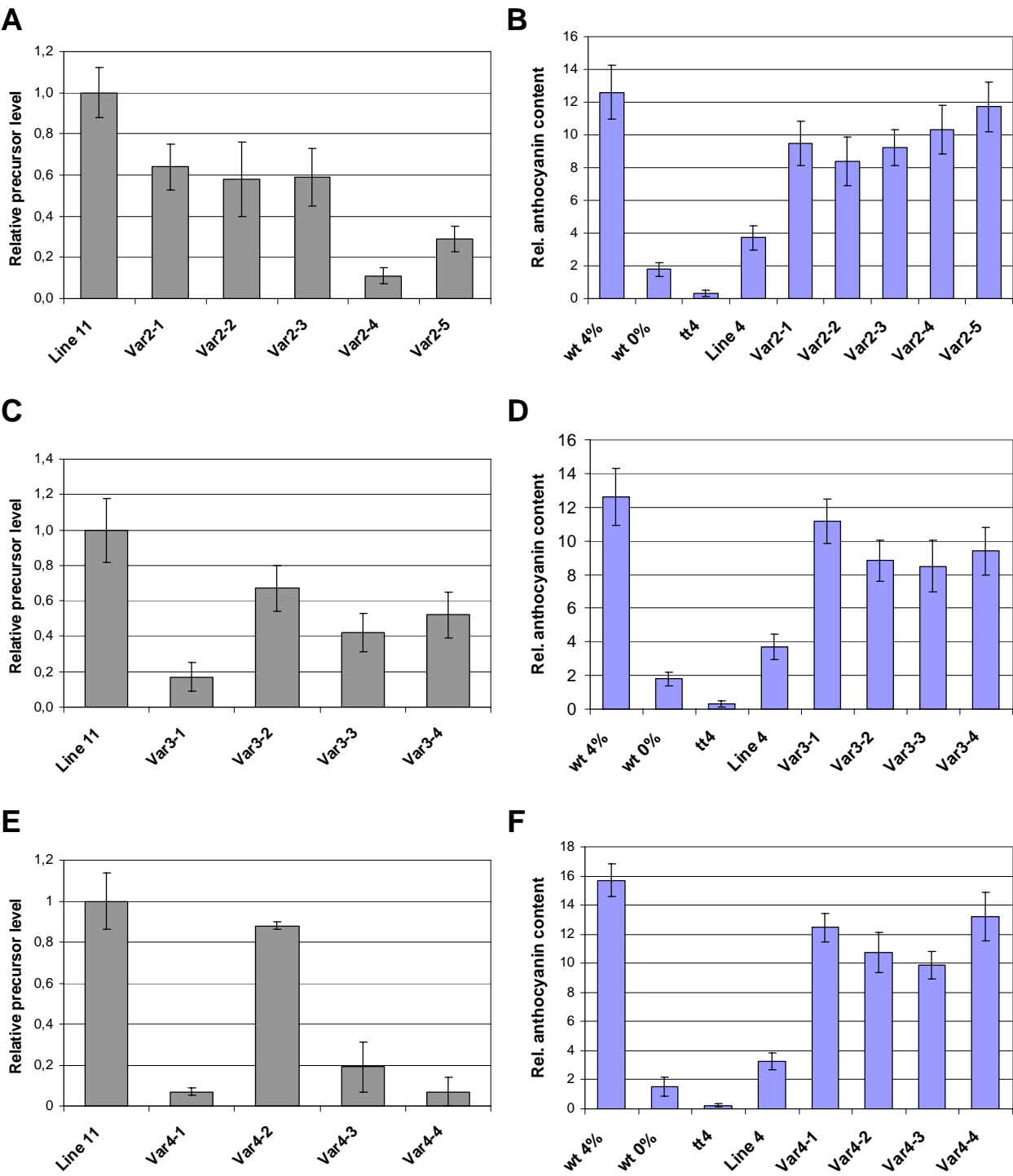

G

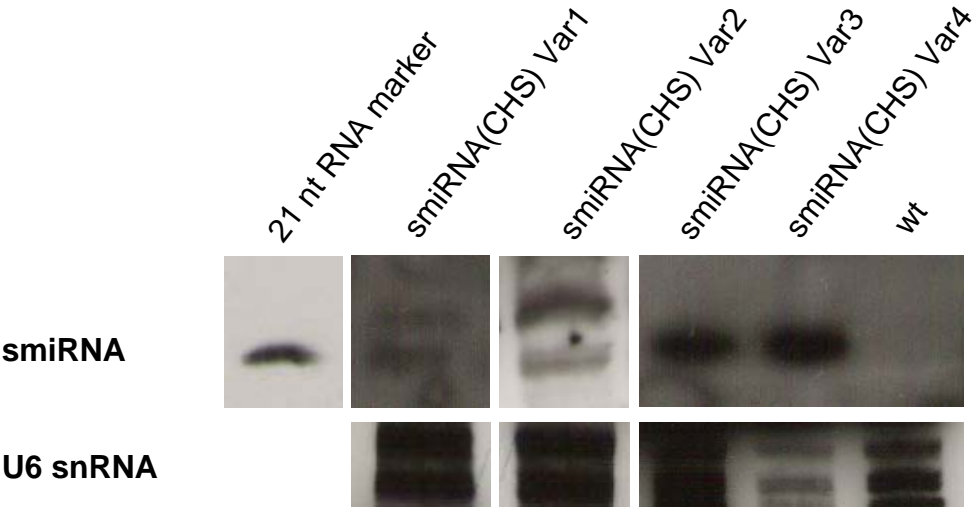

Supplement: Additional file 5 — Molecular and phenotypic analyses of transgenic lines expressing mutant variants Var2, Var3 and Var4 of smiRNA(CHS) ECV. RNA was extracted from different transgenic lines, and qRT-PCR experiments were performed to quantify the relative transcript levels (A, C, E) of the smiRNA(CHS) ECV Var2, Var3 and Var4 precursors. smiRNA(CHS) line 11 (Line 11; see Figures 2 and 3) was used for normalization. (B, D, F) Relative anthocyanin content of the same transgenic lines. Wild type seedlings were grown either without (wt 0%) or with 4% sucrose (wt 4%). All other plants were grown with 4% sucrose. chs knockout (tt4) and smiRNA(CHS) line 4 (Line 4; see Figures 2 and 3) seedlings were measured for comparison. (G) RNA was extracted from transgenic lines expressing pri-smiRNA(CHS) Var1-4 and used for small RNA Northern blots to detect smiRNA production. RNA from wild type (wt) plants served as control. The upper panel shows signals obtained with the smiRNA probe, the signals in the lower panel were obtained using a probe for U6snRNA as loading control. Size marker (M): 21 nt-long RNA oligonucleotide. [file 1756-0500-3-59-S5.PDF]
