# Supplementary material for: Improvement of the design and generation of highly specific plant knockdown lines using primary synthetic microRNAs (pri-smiRNAs)
Source: BMC Res Notes. 2010 Mar 4;3:59. doi: 10.1186/1756-0500-3-59 (PMC2845148; doi:10.1186/1756-0500-3-59)
Supplement: Additional file 6 — Oligo nucleotide sequences. [file 1756-0500-3-59-S6.PDF]

Primers for qRT-PCR experiments

Target *CHS*

Forward primer	5’-GTC TGC CGC TCA GAC CAT
Reverse primer	5’-CGC TTC GTC TAG ACT CTT CAC A

Off-target *At1g49390*

Forward primer	5’-GGA GAG ATA TCT CCA TGC ACC
Reverse primer	5’-CTT CTT GAC CGT CTA CAG AGG

Reference 18S rRNA

Forward primer	5’-TGG GAT AAC ATC ATA GGA TTT CG
Reverse primer	5’-CGA CTG TCC CTG TTA ATC ATT ACT C

Validation experiments using RNA linker-mediated 5’RACE

RNA oligo	5’-GCU GAU GGC GAU GAA UGA ACA CUG CGU UUG CUG GCU UUG AUG AAA

Nested PCR primers

Forward 1	5’-GAA CAC TGC GTT TGC TGG CTT TGA TG
Forward 2	5’-GCT GAT GGC GAT GAA TGA ACA CTG
Reverse 1	5’-CAC CCC ACT CCA ACC CTT CTC C
Reverse 2	5’-CGC ACG CGC TCG ACA TGT TT

Primers for cloning of pri-MIR159a

Forward	5’-ATA TTC TAG ACA AGA TAC TTT GTT TTT CGA TAG ATC
Reverse	5’-CCA AGG ATC TTC TCA TCT ACC CGA GGC AGT TGC

Primers for cloning of pri-smiRNA(CHS)

Standard procedure

Vector primers

Forward	5’-CAG GAA ACA GCT ATG ACC ATG
Reverse	5’-GTA AAA CGA CGG CCA GT

Overlap primers 1 (introducing smiRNA* sequence)

Forward	5’-GGT GCC ATA GAG CGA CAT TTG CAT GAG TTG AGC AGG GTA AAG
Reverse	5’-GAA ATG TCG CTC TAT GGC ACC CTT CCA TCG TCA GAT CAA GAT C

Overlap primers 2 (introducing smiRNA sequence)

Forward	5’-CAA ATG TCC GTC TAT GGC ACC CAT CTT CTT TCA CCT TCT CTA
Reverse	5’-GGT GCC ATA GAC GGA CAT TTG GAA GAG TAA AAG CCA TTA AAG GGC

Primers for cloning of pri-smiRNA(CHS) ECV
ECV procedure

original CHS smiRNA	
NheI primer	5'-ATC GGC TAG CTG GAA GGG TGC CAT AGA GCG ACA TTT GCA TGA GTT GAG CAG GGT AAA G
BsrGI primer	5'-GCT ATG TAC AAG AAG ATG GGT GCC ATA GAC GGA CAT TTG GAA GAG TAA AAG CCA TTA AAG GG
Variant 1	mismatch in 5'region of smiRNA(CHS)
NheI primer	5'-GCT ATG TAC AAG AAG ATG GGT GCC ATA GAC GGA CAG TTA GAA GAG TAA AAG CCA TTA AAG GG
BsrGI primer	5'-ATC GGC TAG CTG GAA GGG TGC CAT AGA GCG ACA GTT ACA TGA GTT GAG CAG GGT AAA G
Variant 2	mismatches in 3'region of smiRNA(CHS)
NheI primer	5'-GCT ATG TAC AAG AAG ATG GGA GCT ATA GAC GGA CAT TTA GAA GAG TAA AAG CCA TTA AAG GG
BsrGI primer	5'-ATC GGC TAG CTG GAA GGG AGC TAT AGA GCG ACA TTT ACA TGA GTT GAG CAG GGT AAA G
Variant 3	introduction of 6 G:U base pairs in smiRNA:mRNA hybrid
NheI primer	5'-GCT ATG TAC AAG AAG ATG GGT GCC ATA GAC AAA CAC CCA GAA GAG TAA AAG CCA TTA AAG GG
BsrGI primer	5'-ATC GGC TAG CTG GAA GGG TGC CAT AGA GTA ACA CCC ACA TGA GTT GAG CAG GGT AAA G
Variant 4	introduction of 7 G:U base pairs in smiRNA:mRNA hybrid
NheI primer	5'-GCT ATG TAC AAG AAG ATG AAT ACC ATA AAC AAA CAT TTA GAA GAG TAA AAG CCA TTA AAG GG
BsrGI primer	5'-ATC GGC TAG CTG GAA GAA TAC CAT AAA GTA ACA TTT ACA TGA GTT GAG CAG GGT AAA G

Hybridization probes and size marker for Northern blots

RNA size marker	
U6snRNA	5'-UGG CCC CUG CGC AAG GAU GAA
probes	
U6snRNA	5'-(Biotinyl) TTC ATC CTT GCG CAG GGG CCA
smiRNA(CHS)	5'-(Biotinyl) GGT GCC ATA GAG CGA CAT TTG
Variant 1+2	5'-(Biotinyl) GGT GCT ATA GAC GGA CAT TTG
Variant 3+4	5'-(Biotinyl) GGT GCC ATA GAC AAA CAT TTA
